# Rest and Pain

**Published:** 1909-07-03

**Authors:** 


### Rest and Pain.

Fifty years ago, in the peroration to his classical lectures "On the Influence of Mechanical and Physiological Rest, and the Diagnostic Value of Pain," John Hilton said: "There is no field open to the future inquirer, from which he will reap a richer reward in the benefit of his race and profession, than from the persevering attempt to interpret the purport and true significance of the manifold pains, by which Nature admonishes us of hidden and otherwise imperceptible evils."

It is open to question how far this forecast has been verified by the event. As to the amount done in this field there can be no two opinions. Probably every practising medical man of to-day makes use, consciously or unconsciously, of the results of work for which the names of Mackenzie and Head may stand typical. The expert neurologist to-day has at his command a large-scale and most detailed map of the nervous system to facilitate his orientation of organic lesions, and he is beginning to know something about the anatomical representation of psychical phenomena. We can all talk glibly enough of "McBurney's spot" and "Robson's point," and we know something about the area of origin and irradiation of renal and biliary and gastric pain; but, with increased facilities for the allocation of pain, tenderness and paræsthesiæ to their anatomical correlations, it is doubtful if we have not lost or are not losing the powers of observation and of memory which enabled the older clinicians to anticipate the findings of these latter days. The advance of chemistry has enormously increased our capacity for abolishing pain irrespective of its cause; and, though occasions arise when all the analgesics within and without the pharmacopœia fail to relieve pain, yet for the most part there is nowadays a tendency to think first of the annihilation of pain, and then, if at all, of its significance. It seems to be a dictate of civilisation.

The fact of the matter is, that all the work done on the lines indicated by Hilton has only revealed the extraordinary complexity of the whole subject; and before the bewildering intricacies of the possible anatomical explanations of subjective phenomena like pain, the busy practitioner recoils upon overt probabilities. Then again "rest" is just as much the fundamental principle of all treatment as it was in the days of Hippocrates; but we find ourselves in a period of reaction from the practice that followed upon the invaluable lessons so well crystallised in these very lectures. Splints and reclining boards and the stringent interdiction of exertion, have given place to massage and gymnastics and graduated exercise. We still rely no less upon the *vis medicatrix naturæ*, but we go further; we search the inner mechanism of natural forces of recuperation and stimulate them with all our power. To a conception of the ultimate nature of pain we are probably but little nearer; current ideas of rest are occidental, if not paradoxical; but the conditions of modern life commonly preclude any metaphysical hindrances, whilst anatomy and physiology, those freedmen of Medicine, are similarly prevented from undue domination.

